# Prevalence and Predictive Value of Anemia and Dysregulated Iron Homeostasis in Patients with COVID-19 Infection

**DOI:** 10.3390/jcm9082429

**Published:** 2020-07-29

**Authors:** Rosa Bellmann-Weiler, Lukas Lanser, Robert Barket, Lukas Rangger, Anna Schapfl, Marc Schaber, Gernot Fritsche, Ewald Wöll, Günter Weiss

**Affiliations:** 1 Department of Internal Medicine II, Infectious Disease, Immunology, Rheumatology, Medical University of Innsbruck, 6020 Innsbruck, Austria; rosa.bellmann-weiler@i-med.ac.at (R.B.-W.); lukas.lanser@i-med.ac.at (L.L.); robert.barket@i-med.ac.at (R.B.); lukas.rangger@student.i-med.ac.at (L.R.); gernot.fritsche@i-med.ac.at (G.F.); 2 Department of Internal Medicine, St. Vinzenz Krankenhaus Betriebs GmbH, 6511 Zams, Austria; anna.schapfl@krankenhaus-zams.at (A.S.); marc.schaber@gmx.at (M.S.); ewald.woell@krankenhaus-zams.at (E.W.)

**Keywords:** anemia, iron metabolism, SARS-CoV-2, COVID-19 outcome

## Abstract

Infections with SARS-CoV-2 can result in severe clinical manifestations. As such patients present with systemic inflammation, we studied the prevalence and predictive value of anemia of inflammation (AI) or functional iron deficiency (FID), originating from immune-mediated alterations of iron homeostasis. Within this retrospective analysis of 259 hospitalized patients with COVID-19, we found that, upon admission, 24.7% were anemic, with the majority suffering from AI (68.8%). Anemia was associated with a significantly higher in-hospital mortality (OR 3.729 (95%CI 1.739–7.995), *p* = 0.001) but not an increased frequency of intensive care unit (ICU) admission or need for mechanical ventilation. FID was present in 80.0% of patients upon admission, linked to more advanced inflammation and associated with significantly longer hospital stay. Notably, a ferritin/transferrin ratio > 10 predicted a five-fold higher risk of ICU admission and an eight-fold higher risk of the need for mechanical ventilation. Anemia and alterations of iron homeostasis are highly prevalent in hospitalized COVID-19 patients. Iron metabolism biomarkers and hemoglobin can contribute to risk stratification of patients, as initial anemia is associated with increased mortality, whereas alterations of iron homeostasis with a higher ferritin/transferrin ratio reflect more advanced inflammation and predicts subsequent insufficient pulmonary oxygenation with the need for ICU admission and mechanical ventilation.

## 1. Introduction

Infections with the severe acute respiratory syndrome-related coronavirus 2 (SARS-CoV-2) emerged as a worldwide pandemic, termed as the coronavirus disease 2019 (COVID-19) affecting millions of people [1,2]. While the majority of infections have a mild clinical course, up to 20% of infected patients need to be hospitalized, mainly because of pneumonia, with eventual admission to intensive care units (ICU) and a need for mechanical ventilation [3,4]. Such severe COVID-19 infections are characterized by a hyper-inflammatory state being associated with increased concentrations of inflammation markers such as C-reactive protein (CRP), interleukin-6 (IL-6) but also of ferritin [5,6]. The latter is the major iron storage protein, the expression of which is induced by iron loading or inflammation. In COVID-19 patients, elevated ferritin levels were related to disease severity, development of acute respiratory distress syndrome (ARDS) and death [5,7,8]. Inflammation leads to typical alterations of iron homeostasis hallmarked by increased iron acquisition and retention within macrophages along with reduced intestinal iron absorption [9]. This results in a reduction of circulating iron levels and a reduced availability of the metal for erythropoiesis where it is needed for the production of hemoglobin. Together with cytokine-mediated inhibition of erythropoiesis, shortened erythrocyte half-life and reduced biological activity of the red cell hormone erythropoietin, this results in the development of anemia of inflammation (AI) [10]. This type of anemia is characterized by reduced circulating levels of iron and transferrin, a reduced saturation of transferrin with iron (TfS) while ferritin levels are normal or increased, which contrasts AI with iron deficiency anemia (IDA) where ferritin levels are low and transferrin levels are usually elevated [10,11]. The presence of anemia has been linked to an unfavorable clinical course of many diseases, including infections or inflammatory disorders [12,13,14,15,16]. Thus, we investigated the prevalence and causes of anemia and alterations of iron homeostasis in a cohort of hospitalized patients with COVID-19 upon admission and linked it to the clinical course and outcome from the disease.

## 2. Materials and Methods

### 2.1. Study Population

We retrospectively analyzed the data of 259 patients with PCR-confirmed SARS-CoV-2 infections who needed hospitalization either at the Innsbruck University Hospital (*n* =129) or Hospital St. Vinzenz Zams (*n* = 130) between 25 February and 20 May, 2020. The study conformed to the principles of the Declaration of Helsinki and was approved by the ethics committee of the Medical University of Innsbruck (ethical vote: 1167/2020, approved 24 July 2020).

### 2.2. Outcome Analysis and Measurements

Fatal events, ICU admission and need/duration of invasive ventilation were recorded during the patients’ hospital stay. The event-free survival was defined as the period of time between the hospitalization date and patients’ in-hospital death.

Blood samples were taken from patients at baseline (day 1 ± 1) and were analyzed by fully automated tests in the laboratories of the hospitals, which undergo regular quality control. Laboratory findings were then extracted from the clinical information system.

### 2.3. Classifications of Anemia and Alterations of Iron Homeostasis

Anemia was defined according to the World Health Organization (WHO) as hemoglobin < 130 g/L in men and hemoglobin < 120 g/L in women. We further classified anemia into severe anemia, defined as hemoglobin < 80 g/L, moderate anemia, defined as hemoglobin 80−109 g/L, and mild anemia, defined as hemoglobin 110−129 g/L in men and 110−119 g/L in women. [17] Anemic patients were also classified according to iron status into those with anemia of inflammation (AI; TSAT < 20% with ferritin > 100 µg/L), iron deficiency anemia (IDA; TSAT < 20% with ferritin < 30 µg/L), AI/IDA (TSAT < 20% with ferritin 30−100 µg/L) and unclassified/multifactorial anemia (TSAT > 20%). Iron deficiency (ID) was defined as TSAT < 20% in combination either with serum ferritin < 100 µg/L (absolute ID) or serum ferritin > 100 µg/L (functional ID) [10,11].

### 2.4. Statistical Analysis

We used the Shapiro–Wilk test to analyze for Gaussian distribution. Variables are depicted as n (%) or medians (25th, 75th percentile), since most biomarkers were not normally distributed. Mann–Whitney-U test, Kruskal–Wallis test or Pearson chi-square tests were performed to test for significant differences between groups. Logistic regression analysis was performed to analyze the effects of risk factors on the probability of complications and death. Risk factors that were significant in univariate logistic regression analysis were considered for multivariate logistic regression analysis. Variables that showed no Gaussian distribution were logarithmized with the natural logarithm for the logistic regression analysis. Spearman rank correlation test was used to correlate continuous variables.

All tests were two-tailed and *p*-values < 0.05 were regarded as statistically significant. Statistical analysis was performed using SPSS Statistics Version 25.0 for Macintosh (IBM Corporation, Armonk, NY, USA).

## 3. Results

### 3.1. Patients Characteristics

We retrospectively analyzed 259 hospitalized patients with COVID-19 infection with a median age of 68 years (53−80 years): 157 men and 102 women. Women were significantly older compared to men (74 years vs. 64 years, *p* = 0.015). During a median hospitalization time of 9 days (5–17 days), 32 patients died and 53 patients were transferred to the ICU (of whom 35 patients needed mechanical ventilation). Of the patients who died, 40.6% died in the ICU and 21.9% died while on mechanical ventilation. While death rates did not significantly differ between men and women (14.6% vs. 8.8%, *p* = 0.164), men were more often transferred to ICU (24.8% vs. 13.7%, *p* = 0.030) and needed mechanical ventilation more frequently (18.5% vs. 5.9%, *p* = 0.004) compared to women.

### 3.2. Anemia in Patients with COVID-19 Infection

**Figure 1:**
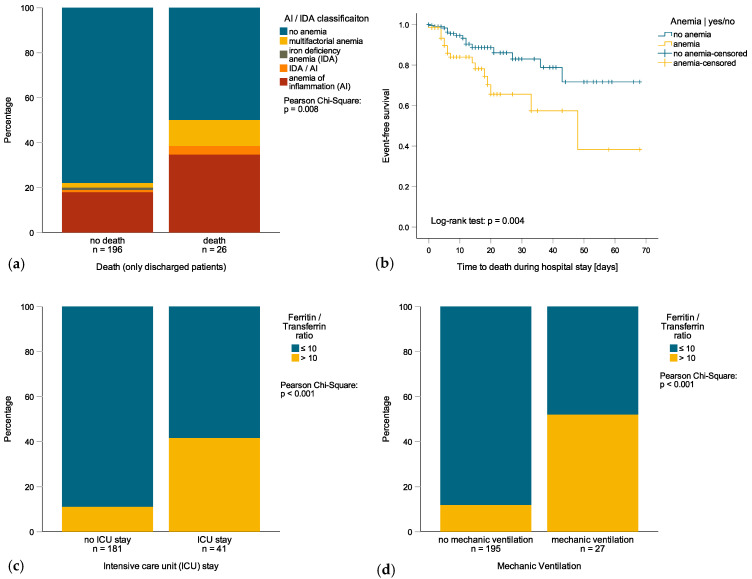
(**a**) prevalence of multifactorial anemia, anemia of inflammation (AI) and/or iron deficiency anemia (IDA) in patients who died or survived; (**b**) Kaplan–Meier survival curve of anemic and non-anemic patients (HR 2.678 (95%CI 1.335 – 5.376), *p* = 0.006); (**c**) prevalence of ferritin/transferrin ratio > 10 vs. ≤ 10 in patients with or without intensive care unit (ICU) admission; (**d**) Prevalence of ferritin/transferrin ratio > 10 vs. ≤ 10 in patients with or without mechanic ventilation.

Among the 259 hospitalized patients with COVID-19 infection, 64 (24.7%) had anemia upon admission. Patients with anemia were older, had a reduced renal function, and had significantly higher levels of inflammatory markers such as CRP or IL-6 (Table 1). Anemia was mild in 38 patients (14.7%), moderate in 21 patients (8.1%) and severe in 5 subjects (1.9%). Most of the anemic patients suffered from AI (68.8%), whereas AI/IDA, IDA and unclassified/multifactorial were less frequent (Figure 1a). Notably, serum iron concentration and transferrin (Tf), but not TfS, were significantly lower in anemic subjects whereas ferritin levels were not different (Table 1). The percentage of patients with anemia progressively increased during hospitalization (68.8% at day 7 of admission). When analyzing the patients’ outcomes, those who were anemic upon admission tended to have a longer hospital stay (Table 1) and a reduced survival rate (Figure 1b). Accordingly, anemia upon admission was associated with a significantly higher mortality during hospitalization (OR 3.729 (95%CI 1.739−7.995), *p* = 0.001, Table 2) even when adjusted for age, comorbidities, eGFR, white blood cells count and PCT levels in logistic regression analysis (OR 5.063 (95%CI 1.260−20.345), *p* = 0.022, Table 2—Multivariate Model I). When differentiating between mild and moderate/serious anemia, only moderate/serious anemia was associated with a significantly higher mortality during hospitalization in multivariate logistic regression analysis (OR 13.323 (95%CI 2.139−82.999), *p* = 0.006, Table 2—Multivariate Model II). Interestingly, no significant correlations were found between initial anemia and the risk of ICU admission (OR 0.867 (95%CI 0.424−1.774), *p* = 0.696, Table 3) or the need for mechanical ventilation (OR 1.259 (95%CI 0.569−2.788), *p* = 0.570, Table S1).

### 3.3. Disturbances of Iron Homeostasis in Patients with COVID-19 Infection

Iron metabolism biomarkers were available from 222 patients. Upon admission, 194 patients (88.2%) had an abnormal iron homeostasis, and the majority of those had functional iron deficiency (FID, *n* = 176, 79.3%; Table S2). The presence of FID was associated with significantly poorer clinical conditions and a longer hospital stay, but was not linked to an increased risk of in-hospital death, ICU admission or the need for mechanical ventilation (Table 2 and Table 3 and Table S1). When analyzing variables of iron homeostasis in terms of patient outcomes, no significant relations were found between mortality rate and elevated ferritin levels, low Tf levels, or low TfS (Table 2). However, elevated ferritin levels were associated with longer hospital stays (rs = 0.251, *p* < 0.001), an increased risk for ICU admission (OR 2.780 (95%CI 1.874−4.124), *p* < 0.001, Table 3) and the need for mechanical ventilation (OR 3.497 (95%CI 2.124−5.757), *p* < 0.001, Table S1). In addition, low Tf levels upon admission but not a low TfS were associated with a longer hospital stay (rs = −0.286, *p* < 0.001), an increased risk for ICU admission (OR 0.059 (95%CI 0.015−0.226), *p* < 0.001, Table 3) and the need for mechanical ventilation (OR 0.079 (95%CI 0.018–0.337), *p* = 0.001, Table S1). Based on the findings of a significant association of either ferritin or transferrin with the clinical course and previous observations indicating that a ferritin/transferrin ratio can discriminate well between true and FID [18] and identify IDA in non-inflammatory subjects with borderline ferritin levels [19], we studied whether a ferritin/transferrin ratio would be an even more powerful predictor of the patients’ clinical course. A ferritin/transferrin ratio > 10 (*n* = 37) well discriminated patients with a higher risk of ICU admission (OR 5.702 (95%CI 2.625–12.388), *p* < 0.001, Figure 1c and for need of mechanical ventilation (OR 8.054 (95%CI 3.369−19.249), *p* < 0.001, Table S1, Figure 1d compared to patients with a ferritin/transferrin ratio ≤ 10 (*n* = 185). An elevated ferritin/transferrin ratio was also associated with longer hospital stays (rs = 0.259, *p* < 0.001).

### 3.4. Inflammation Is Related to Iron Metabolism Biomarkers and Hemoglobin

When calculating for associations between anemia and iron metabolism variables with immune activation upon admission, anemic patients had significantly higher CRP, IL-6 and PCT levels (Table 1). Accordingly, hemoglobin levels were only significantly correlated with CRP levels (rs = −0.163, *p* = 0.009) and tended to be related to IL-6 (rs = −0.135, *p* = 0.067) and PCT levels (rs = −0.124, *p* = 0.083). As expected, patients with AI had the highest CRP (8.69 mg/dL, *p* < 0.001) and IL-6 levels (82.7 ng/L, *p* = 0.007) when compared to patients with IDA (0.12 mg/dL, 3.4 ng/L, respectively) or unclassified/multifactorial anemia (7.79 mg/dL, 70.3 ng/L, respectively).

In addition, patients with FID had significantly higher CRP, IL-6 and PCT levels when compared to patients with absolute ID or no ID (Table S2). Accordingly, ferritin levels positively correlated with CRP (rs = 0.567, *p* < 0.001), IL-6 (rs = 0.552, *p* < 0.001) and PCT levels (rs = 0.442, *p* < 0.001), while Tf levels and the TfS negatively correlated with CRP (Tf: rs = −0.672, *p* < 0.001; TfS: rs = −0.315, *p* < 0.001), IL-6 (Tf: rs = −0.634, *p* < 0.001; TfS: rs = −0.270, *p* = 0.001) and PCT levels (Tf: rs = −0.477, *p* < 0.001; TfS: rs = −0.253, *p* = 0.001). As expected, CRP levels were high significantly correlated to IL-6 levels (rs = 0.757, *p* < 0.001) and PCT levels (rs = 0.677, *p* < 0.001), and IL-6 levels also significantly correlated with PCT levels (rs = 0.650, *p* < 0.001).

## 4. Discussion

Our data indicate that anemia, and specifically AI, is prevalent in patients with severe SARS-CoV-2 infection and that anemia is associated with longer hospital stays, poor clinical conditions and poor survival. This may be linked to reduced tissue oxygenation along with anemia being a reflection of co-morbidities such as impaired renal function or older age [20] or advanced inflammation [10]. It is also possible that multi-morbid patients were more likely to be anemic and less likely to be transferred to ICU when their physical condition deteriorated, a notion which needs to be systemically studied. This would be in line with the observation that only 40.6% of all fatalities occurred on ICU and only 21.9% of the patients who died had been on mechanical ventilation. Moreover, anemic patients had a higher prevalence of comorbidities such as arterial hypertension, cardiovascular disease or chronic kidney disease, all of which are known risk factors for COVID-19-associated death [5]. Moreover, it will be important to perform a future analysis on whether or not pre-existing anemia is already a risk factor for COVID-19 infection and/or a fatal clinical course [21]. Of interest, high ferritin and low transferrin levels were associated with an increased risk for ICU admission and the need for mechanical ventilation. This is in accordance with the association of altered iron homeostasis with more advanced inflammation, the latter promoting lung injury and respiratory failure [22,23]. We introduced a ferritin/transferrin ratio which seems to be a robust and easily available marker for risk stratification at initial presentation of patients in the hospital in terms of ICU admission and the need for mechanical ventilation in patients with COVID-19. FID per se did not result in higher mortality rates, risk for ICU admission, or a need for mechanic ventilation which would be in line with the fact that infection triggered macrophage iron retention as a host defense mechanism to withhold the nutrient iron from invading microbes [24,25,26], thereby possibly reducing the risk of secondary bacterial and fungal infection in those patients, a notion which needs to be prospectively studied. The finding of a close association of ferritin levels with markers of inflammation is in a line with ferritin as an acute phase protein which is induced by several cytokines such as interferon-gamma or IL-6, two major drivers of macrophage activation and lung injury [22]. However, in contrast to other studies, higher ferritin levels were not associated with an increased risk of death [6,7,8]. The association of low transferrin levels with a more severe clinical course also warrants further investigation in order to see whether they may contribute to a more severe course of the disease by preventing detoxification of pro-oxidative free iron in the circulation, thereby contributing to vascular and/or lung tissue injury [27,28,29] or by the inability to limit the access of SARS-CoV-2 to its nutrient iron [30]. Alterations of iron homeostasis may contribute to the pathogenesis of severe COVID-19 infection by mechanisms which need to be disentangled by future research. This also harbors the potential for therapeutic intervention by treatments which modulate iron availability such as application of transferrin, hepcidin-modifying agents or iron chelation. If increased levels of non-transferrin bound iron are found to be associated with tissue damage and a worse outcome, either iron chelators, transferrin or hepcidin agonist may help to reduce iron catalyzed radical formation and cellular damage in organs such as the lung or the vasculature. Furthermore, once the impact of alterations of iron availability on adaptive and innate immune functions as well as on viral pathogenicity and replication have been clarified, therapeutic spatiotemporal alterations of iron availability for either the virus or immune cells may favorably affect the course of the infection and associated pathologic inflammation [24,25,26,27,31]. Nonetheless, both anemia and inflammation-induced disturbances of iron homeostasis are important clinical predictors for risk stratification of SARS-CoV-2-infected patients, thereby improving the clinical management specifically of those at the highest risk [32].

### Limitations

This was a retrospective observational analysis of COVID-19 patients with the need for in-hospital treatment in the region of Tyrol. As it is in the nature of a retrospective analysis, these results do not prove any causality. Additionally, we did not have iron metabolism variables available for all patients included in the study, depicting a probable selection bias and resulting in a smaller sample size in the multivariate analysis. Hemoglobin as a continuous variable was not a significant predictor in multivariate regression analysis, depicting the possibility of residual confounding for anemia classification. Finally, regional economic/social conditions must be taken into account when interpreting the results, which is why the findings do not allow unrestricted generalization for all COVID-19 patients. 

## 5. Conclusions

The results of our study indicate that anemia, and specifically AI, is prevalent in patients with severe COVID-19 disease and is associated with an unfavorable outcome. We also introduced a ferritin/transferrin ratio which seems to be a robust and easily available marker for risk stratification at hospital admission of patients with SARS-CoV-2 infection. A higher ferritin/transferrin ratio reflects more advanced inflammation and predicts subsequent insufficient pulmonary oxygenation with the need for ICU admission and mechanical ventilation. Further studies are needed to investigate probable (direct) effects of changed iron homeostasis on the pathogenesis and severity of COVID-19 infections, as well as the potential for therapeutic interventions by modulating iron availability.

## Figures and Tables

**Table 1:** Patients’ characteristics of anemic patients at hospital admission. *

	No Anemia	Anemia	
	*n* = 195	*n* = 64	
	Median (IQR) or *n* (%)	Median (IQR) or *n* (%)	*p*-Value ^1^
Demographic Characteristics			
Age (Years)	63 (51–77)	78 (65–86)	<0.001
BMI (kg/m^2^)	26.12 (23.79–28.73)	26.00 (22.10–28.03)	0.233
Sex, Men	119 (61.0%)	38 (59.4%)	0.815
Clinical Characteristics			
Temperature (°C)	37.0 (36.2–37.9)	36.9 (36.3–38.0)	0.803
SpO2 (%)	95 (92–96)	94 (90–95)	0.012
O2 Requirement (L)	0 (0–1)	0 (0–2)	0.126
Hospitalization (Days) ^2^	9 (5–15)	12 (7–20)	0.062
ICU Admission	41 (21.0 %)	12 (18.8%)	0.695
ICU Duration (Days) ^2,3^	18 (7–32)	24 (20–41)	0.309
Mechanical Ventilation	25 (12.8%)	10 (15.6%)	0.569
Mechanical Ventilation Duration (Days) ^2,3^	16 (14–21)	16 (12–24)	0.882
Death During Hospital Stay	16 (8.2%)	16 (25.0%)	<0.001
Comorbidities and Risk Factors			
Cardiovascular Disease (CVD)	104 (53.3%)	48 (75.0%)	0.002
Arterial Hypertension	86 (44.1%)	38 (59.4%)	0.034
Coronary Artery Disease (CAD)	23 (11.8%)	12 (18.8%)	0.158
Chronic Heart Failure (CHF)	5 (2.6%)	4 (6.3%)	0.162
Diabetes Mellitus (DM)	31 (15.9%)	14 (21.9%)	0.273
Chronic Kidney Disease (CKD)	4 (2.1%)	11 (17.2%)	<0.001
Malignancies	14 (7.2%)	7 (10.9%)	0.339
Chronic Obstructive Pulmonary Disease	21 (10.8%)	2 (3.1%)	0.062
Bronchial Asthma	8 (4.1%)	1 (1.6%)	0.336
Nicotine Abuse, Actual or Former	29 (16.6%)	5 (8.5%)	0.286
Laboratory Findings			
eGFR (mL/min)	77.9 (56.5–93.8)	64.1 (42.1–85.7)	0.005 (*n* = 257)
AST (U/L)	34 (26–49)	36 (23–49)	0.727 (*n* = 253)
ALT (U/L)	25 (18–42)	19 (14–34)	0.006 (*n* = 245)
AP (U/L)	67 (51–89)	69 (55–87)	0.620 (*n* = 241)
MCV (fl)	88.2 (85.0–91.3)	89.0 (85.5–93.6)	0.313 (*n* = 252)
MCH (pg)	30.1 (29.0–31.2)	30.3 (28.3–31.1)	0.859 (*n* = 252)
Hemoglobin (g/L)	141 (134–153)	113 (101–121)	<0.001 (*n* = 259)
Hematocrit (L/L)	0.420 (0.394–0.450)	0.340 (0.307–0.360)	<0.001 (*n* = 256)
Thrombocytes (G/L)	191 (151–263)	201 (157–265)	0.834 (*n* = 256)
Iron (μmol/L)	5.0 (3.7–8.5)	3.8 (3.0–6.3)	0.001 (*n* = 226)
Ferritin (μg/L)	566 (233–1 243)	545 (219–954)	0.486 (*n* = 241)
Transferrin (mg/dL)	191 (162–230)	154 (129–183)	<0.001 (*n* = 224)
Ferritin/Transferrin ratio ^4^	2.88 (1.03–6.22)	3.60 (1.33–9.03)	0.263 (*n* = 222)
TSAT (%)	11 (8–16)	12 (7–18)	0.954 (*n* = 224)
Leukocytes (G/L)	6.00 (4.68–7.98)	5.89 (4.00–8.00)	0.605 (*n* = 256)
CRP (mg/dL)	3.42 (0.96–8.17)	8.05 (3.47–15.46)	<0.001 (*n* = 255)
IL-6 (ng/L)	32.8 (15.3–76.3)	79.0 (22.8–172.8)	0.002 (*n* = 186)
PCT (ng/mL)	0.08 (0.06–0.22)	0.18 (0.09–0.51)	0.002 (*n* = 195)

* IQR = interquartile range; BMI = body mass index; SpO2 = peripheral capillary oxygen saturation; O2 = oxygen; ICU = intensive care unit; eGFR = estimated glomerular filtration rate; AST = aspartate aminotransferase; ALT = alanine aminotransferase; AP = alkaline phosphatase; MCV = mean corpuscular volume; MCH = mean corpuscular hemoglobin; TSAT = transferrin saturation; CRP = C-reactive protein; IL-6 = interleukin 6; PCT = procalcitonin; ^1^ *p*-Value was calculated with the Mann–Whitney U test or Pearson Chi-Square test and n depicts the number of patients with each laboratory finding available; ^2^ without patients who died during hospital stay; ^3^ patients with ICU stay only; ^4^ calculated as a ratio of ferritin (µg/L)/transferrin (mg/dL).

**Table 2:** Logistic Regression Analysis in terms of death during hospitalization (yes/no). *

	Logistic Regression Analysis								
	Univariate Model			Multivariate Model I			Multivariate Model II		
	OR	95% CI	*p*-Value	OR	95% CI	*p*-Value	OR	95 % CI	*p*-Value
Demographic Characteristics									
Age (years)	1.083	1.046–1.122	<0.001	1.045	0.974–1.123	0.221	1.049	0.976–1.128	0.193
BMI (kg/m^2^) ^1^	0.803	0.173–3.730	0.779						
Sex, Women vs. Men	0.564	0.250–1.273	0.168						
Clinical Characteristics									
Temperature (° C)	1.091	0.736–1.619	0.664						
SpO2 (%)	0.952	0.884–1.025	0.190						
O2 Requirement (L) ^1^	1.864	0.854–4.070	0.118						
Comorbidities									
CVD, yes vs. no	5.815	1.975–17.117	0.001	2.127	0.309–14.647	0.443	2.532	0.341–18.798	0.364
DM, yes vs. no	1.543	0.513–4.639	0.440						
CKD, yes vs. no	10.476	3.493–31.422	<0.001						
COPD, yes vs. no	2.851	1.032–7.874	0.043	5.064	0.867–29.563	0.072	4.701	0.711–31.077	0.108
Laboratory Findings									
eGFR (mL/min)	0.958	0.943–0.974	<0.001	0.965	0.935–0.996	0.026	0.965	0.934–0.996	0.027
Iron (μmol/L) ^1^	0.656	0.306–1.406	0.279						
Ferritin (μg/L) ^1^	0.957	0.688–1.332	0.794						
Transferrin (mg/dL) ^1^	0.223	0.054–0.924	0.039						
Ferritin/Transferrin ratio ^1,2^	1.021	0.767–1.360	0.885						
TSAT (%) ^1^	1.038	0.473–2.277	0.926						
Leukocytes [G/L] ^1^	4.879	2.201–10.814	<0.001	8.576	2.550–28.846	0.001	11.099	2.914–42.276	<0.001
CRP (mg/dL) ^1^	1.374	1.041–1.814	0.025						
IL-6 (ng/L) ^1^	1.415	1.046–1.915	0.024						
PCT (ng/mL) ^1^	1.475	1.082–2.010	0.014	1.516	0.907–2.535	0.113	1.516	0.989–2.560	0.119
Classifications									
Anemia									
Anemia vs. no Anemia	3.729	1.739–7.995	0.001	5.063	1.260–20.345	0.022			
WHO Classification									
Mild Anemia vs. no Anemia	2.983	1.174–7.581	0.022				2.977	0.607–14–608	0.179
Moderate/Severe anemia vs. no Anemia	4.972	1.871–13.213	0.001				13.323	2.139–82.999	0.006
Iron Deficiency									
Absolute ID vs. no ID	0.458	0.082–2.572	0.375						
Functional ID vs. no ID	0.418	0.150–1.165	0.095						

* Multivariate model I analyses the predictive value of anemia, while the multivariate model II analyses the predictive value of anemic subgroups. Because of the high significantly correlation of PCT levels with CRP and IL-6 levels, only PCT was included into the multivariate model since it showed the highest predictive value in the univariate analysis. The chi-square test was 64.621 (*p* < 0.001) for multivariate model I (*n* = 194—only patients with all included variables were available) and 67.581 (*p* < 0.001) for multivariate model II (*n* = 194—only patients with all included variables were available). OR = odds ratio; CI = confidence interval; BMI = body mass index; SpO2 = peripheral capillary oxygen saturation; O2 = oxygen; CVD = cardiovascular disease; DM = diabetes mellitus; CKD = chronic kidney disease; COPD = chronic obstructive pulmonary disease; eGFR = estimated glomerular filtration rate; TSAT = transferrin saturation; CRP = C-reactive protein; IL-6 = interleukin 6; PCT = procalcitonin; WHO = World Health Organization; ID = iron deficiency. ^1^ logarithmized with the natural logarithm because not normally distributed; ^2^ calculated as a ratio of ferritin (µg/L)/transferrin (mg/dL).

**Table 3:** Logistic Regression Analysis in terms of risk for ICU admission (yes/no). *

	Logistic Regression Analysis								
	Univariate Model		Multivariate Model I			Multivariate Model II			
	OR	95% CI	*p*-Value	OR	95% CI	*p*-Value	OR	95% CI	*p*-Value
Demographic Characteristics									
Age (Years)	0.988	0.972–1.005	0.162						
BMI (kg/m^2^) ^2^	1.243	0.267–5.791	0.781						
Sex, Women vs. Men	0.481	0.246–0.941	0.032	10.348	1.911–56.032	0.007	5.139	1.213–21.777	0.026
Clinical Characteristics									
Temperature (° C)	1.741	1.258–2.408	0.001	1.568	0.939–2.617	0.085	1.551	0.958–2.512	0.074
SpO2 (%)	0.914	0.861–0.970	0.003	0.857	0.754–0.974	0.018	0.896	0.801–1.003	0.057
O2 Requirement (L) ^1^	3.699	1.870–7.318	<0.001						
Comorbidities									
CVD, yes vs. no	1.643	0.867–3.113	0.128						
DM, yes vs. no	2.639	1.303–5.346	0.007	2.602	0.726–9.319	0.142	3.105	0.913–10.555	0.070
CKD, yes vs. no	1.447	0.442–4.740	0.542						
COPD, yes vs. no	2.805	1.141–6.894	0.025	11.685	2.078–65.703	0.005	6.734	1.406–32.260	0.017
Laboratory Findings									
eGFR (mL/min)	1.003	0.992–1.015	0.563						
Iron (μmol/L) ^1^	0.406	0.203–0.812	0.011						
Ferritin (μg/L) ^1^	2.780	1.874–4.124	<0.001	4.084	1.599–10.431	0.003			
Transferrin (mg/dL) ^1^	0.059	0.015–0.226	<0.001	3.424	0.339–34.535	0.297			
Ferritin/Transferrin Ratio ^1,2^	2.265	1.598–3.210	<0.001				2.081	1.089–3.976	0.026
TSAT (%) ^1^	0.955	0.499–1.829	0.889						
Leukocytes (G/L) ^1^	3.393	1.762–6.535	<0.001	3.518	0.930–13.314	0.064	3.927	1.210–12.743	0.023
CRP (mg/dL) ^1^	1.798	1.373–2.353	<0.001						
IL-6 (ng/L) ^1^	2.224	1.632–3.031	<0.001	1.961	1.088–3.535	0.025	1.646	0.968–2.800	0.066
PCT (ng/mL) ^1^	1.111	0.869–1.421	0.400						
Classifications									
Anemia									
Anemia vs. no Anemia	0.867	0.424–1.774	0.696						
WHO Classification									
Mild Anemia vs. no anemia	1.166	0.512–2.656	0.715						
Moderate/Severe Anemia vs. no Anemia	0.490	0.140–1.713	0.264						
Iron Deficiency									
Absolute ID vs. no ID	0.147	0.017–1.297	0.084						
Functional ID vs. no ID	0.556	0.225–1.373	0.203						

* Multivariate model I analyses the predictive value of ferritin and transferrin levels on their own, while the multivariate model II analyses the predictive value of the combined ferritin/transferrin ratio. Because of the high significant correlation of CRP and IL-6 levels, only IL-6 was included into the multivariate model since it showed the highest predictive value in the univariate analysis. The chi-square test was 54.817 (*p* < 0.001) for multivariate model I *(n* = 139—only patients with all included variables available) and 48.315 (*p* < 0.001) for multivariate model II (*n* = 140—only patients with all included variables available). OR = odds ratio; CI = confidence interval; BMI = body mass index; SpO2 = peripheral capillary oxygen saturation; O2 = oxygen; CVD = cardiovascular disease; DM = diabetes mellitus; CKD = chronic kidney disease; COPD = chronic obstructive pulmonary disease; eGFR = estimated glomerular filtration rate; TSAT = transferrin saturation; CRP = C-reactive protein; IL-6 = interleukin 6; PCT = procalcitonin; WHO = World Health Organization; ID = iron deficiency. ^1^ logarithmized with the natural logarithm because not normally distributed; ^2^ calculated as a ratio of ferritin (µg/L)/transferrin (mg/dL).

## Supplementary Materials

The following are available online at https://www.mdpi.com/2077-0383/9/8/2429/s1, Table S1: Logistic Regression Analyses in terms of need for mechanical ventilation (yes/no), Table S2: Patients’ characteristics of patients with ID at hospital admission.
