# Supplementary material for: Prevalence and Predictive Value of Anemia and Dysregulated Iron Homeostasis in Patients with COVID-19 Infection
Source: J Clin Med. 2020 Jul 29;9(8):2429. doi: 10.3390/jcm9082429 (PMC7464087; doi:10.3390/jcm9082429)
Supplement: Supplementary file 1 [file jcm-09-02429-s001.pdf]

Supplementary materials

Prevalence and predictive value of anemia and dysregulated iron homeostasis in patients with COVID-19 infection

Rosa Bellmann-Weiler [1], Lukas Lanser [1], Robert Barket [1], Lukas Rangger [1], Anna Schapfl [2], Marc Schaber [2], Gernot Fritsche [1], Ewald Wöll [2], Günter Weiss [1,*]

[1] Department of Internal Medicine II, Infectious Disease, Immunology, Rheumatology, Medical University of Innsbruck, Innsbruck, Austria

[2] Department of Internal Medicine, St. Vinzenz Krankenhaus Betriebs GmbH, Zams, Austria

* Correspondence: guenter.weiss@i-med.ac.at; Tel.: +43-512-504-23251 (G.W.)

Table S1. Logistic Regression Analysis in terms of need for mechanical ventilation (yes/no). *

	Logistic Regression Analysis								
	Univariate Model			Multivariate Model I			Multivariate Model II		
	OR	95 % CI	p-Value	OR	95 % CI	p-Value	OR	95 % CI	p-Value
Demographic characteristics									
Age [years]	0.988	0.969 - 1.008	0.238						
BMI [kg/m^2] [1]	1.335	0.206 - 8.666	0.762						
Sex, women *vs.* men	0.276	0.110 - 0.691	0.006	1.679	0.297 - 9.488	0.558	1.563	0.289 - 8.465	0.604
Clinical characteristics									
Temperature [°C]	1.844	1.238 - 2.747	0.003	1.671	0.931 - 2.998	0.085	1.822	1.032 - 3.215	0.039
SpO2 [%]	0.884	0.825 - 0.946	< 0.001	0.859	0.763 - 0.967	0.012	0.875	0.781 - 0.981	0.022
O2 requirement [L] [1]	3.939	1.904 - 8.146	< 0.001						
Comorbidities									
CVD, yes *vs.* no	1.909	0.876 - 4.163	0.104						
DM, yes *vs.* no	3.020	1.371 - 6.652	0.006	3.523	0.929 - 13.355	0.012	3.658	0.974 - 13.743	0.055
CKD, yes *vs.* no	0.984	0.212 - 4.558	0.983						
COPD, yes *vs.* no	1.907	0.659 - 5.518	0.233						
Laboratory findings									
eGFR [mL/min]	1.003	0.989 - 1.016	0.721						
Iron [μmol/L] [1]	0.390	0.169 - 0.899	0.027						
Ferritin [μg/L] [1]	3.497	2.124 - 5.757	< 0.001	4.570	1.664 - 12.551	0.003			
Transferrin [mg/dL] [1]	0.079	0.018 - 0.337	0.001	5.658	0.435 - 73.544	0.185			
Ferritin/Transferrin ratio [1, 2]	2.768	1.766 - 4.340	< 0.001				2.688	1.215 - 5.946	0.015
TSAT [%] [1]	0.967	0.447 - 2.091	0.931						
Leukocytes [G/L] [1]	2.134	1.045 - 4.357	0.037	0.685	0.174 - 2.704	0.589	0.738	0.197 - 2.755	0.651
CRP [mg/dL] [1]	2.729	1.797 - 4.143	< 0.001	1.775	0.772 - 4.084	0.177	1.449	0.699 - 3.004	0.319
IL-6 [ng/L] [1]	2.143	1.557 - 2.949	< 0.001						
PCT [ng/mL] [1]	1.302	0.989 - 1.716	0.060						
Classifications									
Anemia									
anemia *vs.* no anemia	1.259	0.569 - 2.788	0.570						

	Logistic Regression Analysis								
	Univariate Model			Multivariate Model I			Multivariate Model II		
	OR	95 % CI	p-Value	OR	95 % CI	p-Value	OR	95 % CI	p-Value
WHO classification									
mild anemia *vs.* no anemia	1.813	0.748 - 4.397	0.188						
moderate/severe anemia *vs.* no anemia	0.567	0.126 - 2.546	0.459						
Iron deficiency									
absolute ID *vs.* no ID	0.271	0.029 - 2.533	0.252						
functional ID *vs.* no ID	0.623	0.214 - 1.815	0.386						

* Multivariate model I analyses the predictive value of ferritin and transferrin levels by their own, while the multivariate model II analyses the predictive value of the combined ferritin/transferrin ratio. Because of the high significantly correlation of PCT, CRP and IL-6 levels, only CRP was included into the multivariate model since it showed the highest predictive value in the univariate analysis. The chi-square test was 43.446 ($p < 0.001$) for multivariate model I (n = 192 – only patients with all included variables available) and 39.538 ($p < 0.001$) for multivariate model II (n = 192 – only patients with all included variables available). OR = odds ratio; CI = confidence interval; BMI = body mass index; SpO2 = peripheral capillary oxygen saturation; O2 = oxygen; CVD = cardiovascular disease; DM = diabetes mellitus; CKD = chronic kidney disease; COPD = chronic obstructive pulmonary disease; eGFR = estimated glomerular filtration rate; TSAT = transferrin saturation; CRP = C-reactive protein; IL-6 = interleukin 6; PCT = procalcitonin; WHO = World Health Organization; ID = iron deficiency. [1] logarithmized with the natural logarithm because not normally distributed; [2] calculated as a ratio of ferritin (µg/L) / transferrin (mg/dL)

Table S2. Patients' characteristics of patients with ID at hospital admission. *

	no ID	absolute ID	functional ID	
	n = 28	n = 18	n = 176	
	Median (IQR)	Median (IQR)	Median (IQR)	p-Value [1]
Demographic characteristics				
Age [years]	61 (52 - 76)	54 (36 - 78)	71 (55 - 80)	0.054
**BMI [kg/m²]**	**25.46 (21.69 - 28.10)**	**21.90 (21.34 - 25.90)**	**26.32 (24.22 - 28.63)**	**0.021**
**Sex [men]**	**18 (64.3 %)**	**2 (11.1 %)**	**115 (65.3 %)**	**< 0.001**
Clinical characteristics				
**Temperature [°C]**	**36.1 (35.9 - 37.0)**	**36.8 (36.2 - 37.2)**	**37.2 (36.4 - 38.0)**	**0.001**
**SpO2 [%]**	**95 (93 - 97)**	**98 (95 - 99)**	**94 (90 - 96)**	**< 0.001**
O2 requirement [L]	0 (0 - 0)	0 (0 - 0)	0 (0 - 2)	0.052
**Hospitalization [days] [2]**	**6 (3 - 9)**	**7 (2 - 10)**	**10 (6 - 17)**	**0.002**
ICU admission	8 (28.6 %)	1 (5.6 %)	32 (18.2 %)	0.142
ICU duration [days] [2, 3]	16 (1 - 29)	(-)	23 (12 - 32)	0.367
Mechanical ventilation	5 (17.9 %)	1 (5.6 %)	21 (11.9 %)	0.451
Mechanical ventilation duration [days] [2, 3]	14 (10 - 15)	(-)	15 (12 - 18)	0.313
Death during hospital stay	6 (21.4 %)	2 (11.1 %)	18 (10.2 %)	0.230
Duration till death [days] [4]	6 (5 - 15)	16 (4 - 27)	7 (5 - 14)	0.977
Comorbidities and risk factors				
Cardiovascular disease	16 (57.1 %)	8 (44.4 %)	108 (61.4 %)	0.366
**Arterial hypertension**	**10 (35.7 %)**	**5 (27.8 %)**	**93 (52.8 %)**	**0.044**
Coronary artery disease	3 (10.7 %)	0 (0.0 %)	29 (16.5 %)	0.139
**Chronic heart failure**	**2 (7.1 %)**	**2 (11.1 %)**	**3 (1.7 %)**	**0.041**
Diabetes mellitus	4 (14.3 %)	1 (5.6 %)	31 (17.6 %)	0.399
**Chronic kidney disease**	**4 (14.3 %)**	**3 (16.7 %)**	**8 (4.5 %)**	**0.035**
Malignancies	0 (0.0 %)	3 (16.7 %)	17 (9.7 %)	0.126

	no ID	absolute ID	functional ID	
	n = 28	n = 18	n = 176	
	Median (IQR)	Median (IQR)	Median (IQR)	p-Value [1]
COPD	4 (14.3 %)	1 (5.6 %)	15 (8.5 %)	0.532
Bronchial asthma	0 (0.0 %)	0 (0.0 %)	8 (4.5 %)	0.338
Nicotine abuse, actual / former	4 (14.3 %)	1 (7.1 %)	25 (15.7 %)	0.623
Laboratory findings				
eGFR [mL/min]	79.7 (56.0 - 96.9)	53.9 (41.0 - 91.0)	75.4 (54.1 - 88.2)	0.258
MCV [fl]	88.1 (86.6 - 95.2)	87.5 (83.7 - 91.0)	88.3 (84.9 - 91.3)	0.391
MCH [pg]	29.9 (28.9 - 31.5)	28.9 (27.7 - 31.0)	30.2 (29.0 - 31.1)	0.112
Hemoglobin [g/L]	137 (122 - 154)	130 (112 - 139)	136 (126 - 146)	0.085
Hematocrit [L/L]	0.405 (0.365 - 0.453)	0.392 (0.330 - 0.411)	0.400 (0.363 - 0.439)	0.302
Thrombocytes [G/L]	250 (190 - 302)	227 (172 - 252)	190 (153 - 262)	0.069
**Iron [μmol/L]**	**11.7 (8.5 - 14.3)**	**8.3 (3.8 - 10.6)**	**4.2 (3.4 - 5.7)**	**< 0.001**
**Ferritin [μg/L]**	**625 (193 - 1.563)**	**57 (31 - 71)**	**582 (295 - 1.155)**	**< 0.001**
**Transferrin [mg/dL]**	**176 (147 - 230)**	**284 (249 - 303)**	**181 (150 - 207)**	**< 0.001**
**Ferritin/Transferrin ratio [5]**	**4.14 (0.78 - 11.30)**	**0.21 (0.10 - 0.26)**	**3.41 (1.49 - 7.48)**	**< 0.001**
**TSAT [%]**	**25 (22 - 32)**	**12 (5 - 16)**	**10 (7 - 14)**	**< 0.001**
Leukocytes [G/L]	6.30 (5.40 - 8.38)	4.61 (3.60 - 7.94)	6.00 (4.67 - 7.76)	0.129
**CRP [mg/dL]**	**1.34 (0.11 - 7.80)**	**0.46 (0.10 - 2.38)**	**4.66 (2.00 - 12.29)**	**< 0.001**
**IL-6 [ng/L]**	**23.8 (4.6 - 36.9)**	**3.5 (2.0 - 15.1)**	**53.0 (21.8 - 107.5)**	**< 0.001**
**PCT [ng/mL]**	**0.06 (0.00 - 0.32)**	**0.07 (0.00 - 0.08)**	**0.11 (0.07 - 0.25)**	**0.028**

* OR = odds ratio; CI = confidence interval; BMI = body mass index; SpO2 = peripheral capillary oxygen saturation; O2 = oxygen; COPD = chronic obstructive pulmonary disease; eGFR = estimated glomerular filtration rate; TSAT = transferrin saturation; CRP = C-reactive protein; IL-6 = interleukin 6; PCT = procalcitonin. [1] calculated with the Mann-Whitney U test or Pearson Chi-Square test; [2] without patients who died during hospital stay; [3] patients with ICU stay only; [4] patients who died only; [5] calculated as a ratio of ferritin (μg/L) / transferrin (mg/dL)
